# Supplementary material for: LRP1 Deficiency Promotes Mitostasis in Response to Oxidative Stress: Implications for Mitochondrial Targeting after Traumatic Brain Injury
Source: Cells. 2023 May 22;12(10):1445. doi: 10.3390/cells12101445 (PMC10217498; doi:10.3390/cells12101445)
Supplement: Supplementary file 1 [file cells-12-01445-s001.zip › cells-2408460-supplementary.pdf]

Supplemental Methods:

Mito-stress test: Using a Seahorse XFe96 Flux Analyzer (Agilent Technologies, Palo Alto, CA, USA), a mitochondrial stress test was performed according to the manufacturer's instructions. Briefly, WT and LKO cells were seeded at a density of 1× 104 cells per well on XF microplates. After 24hrs the cells were treated with either AAPH or vehicle overnight. Then, the cells were incubated with XF assay medium with substrates (100 µM BSA-palmitate, 100 µM L-Carnitine and 10 mM glucose) or (10 mM glucose, 1 mM pyruvate and 2 mM L-glutamine) for an hour before OCR measurement. After three measurements of baseline OCR, respiratory chain inhibitors were added sequentially into each well as follows; 2.5 µM Oligo, 4 µM FCCP and 0.8/1 µM Rot/Ant. To optimize AAPH concentration, dose response to AAPH was done with 1, 2, 3 and 5 mM. After each inhibitor an addition three OCR readings were taken. Different OCR parameters were calculated by Wave software version (Agilent Technologies) or manually. Final OCR measurements were normalized to cell numbers.

Fig S1:

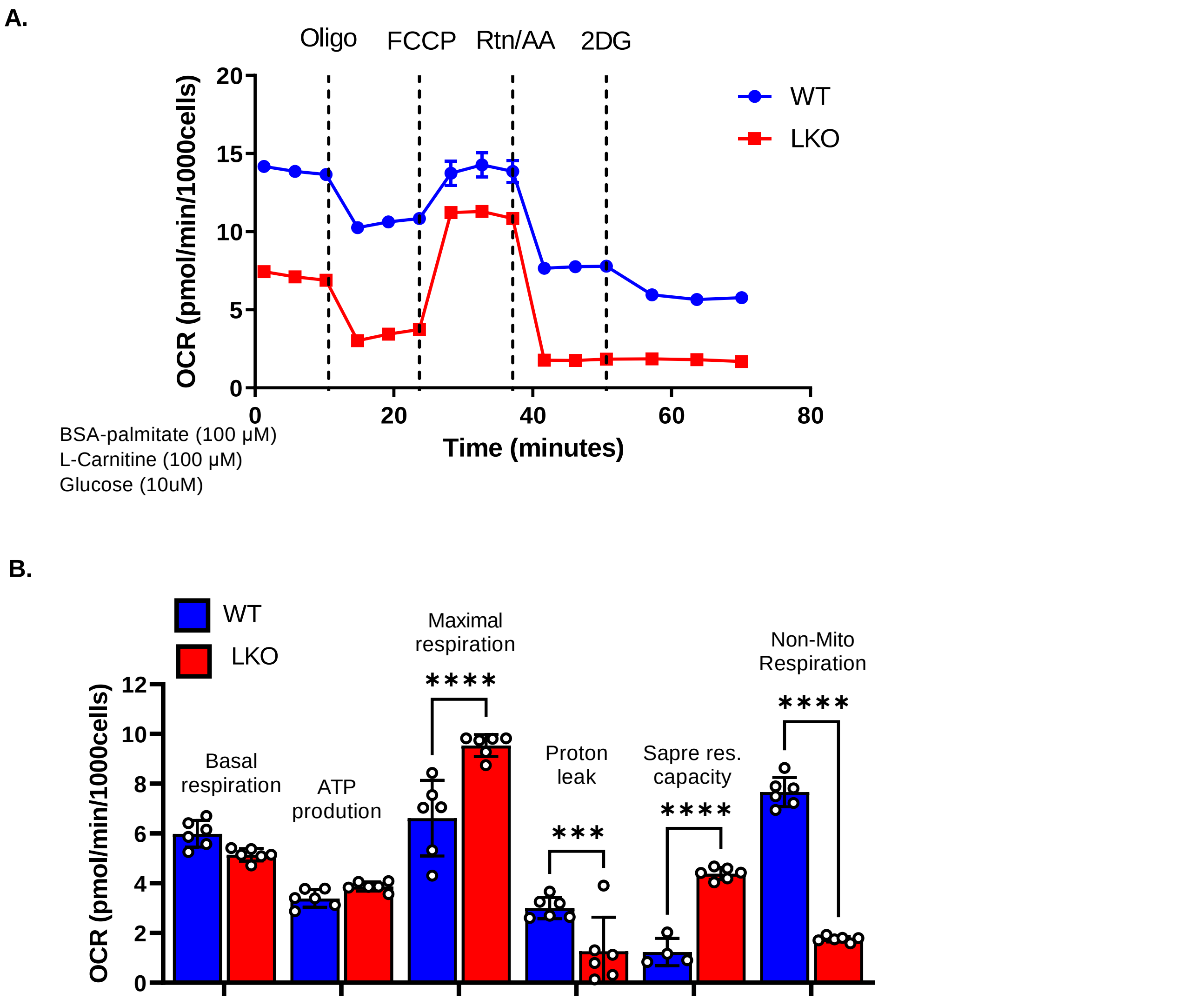

Figure S1. Fatty acid increases mitochondrial bioenergetics in LRP1-deficient cells

A, Traces of mito-stress test (100 µM BSA-palmitate, 100 µM L-Carnitine and 10 mM glucose as substrates) using different respiratory chain inhibitors (oligomycin, FCCP and Rotenone + Antimycin and 2DG) from WT and LKO cells. **B,** Summary of basal respiration, maximal respiration, ATP production, proton leak, spare respiratory capacity and Non-mitochondrial respiration from Seahorse XFe96 respectively (n=6 technical replicates/group). Data represented as Mean ± SD. $P \leq 0.0001$ ****; $P \leq 0.001$ ***; $P \leq 0.01$** by two-way ANOVA with Sidak post-hoc.

Fig S2:

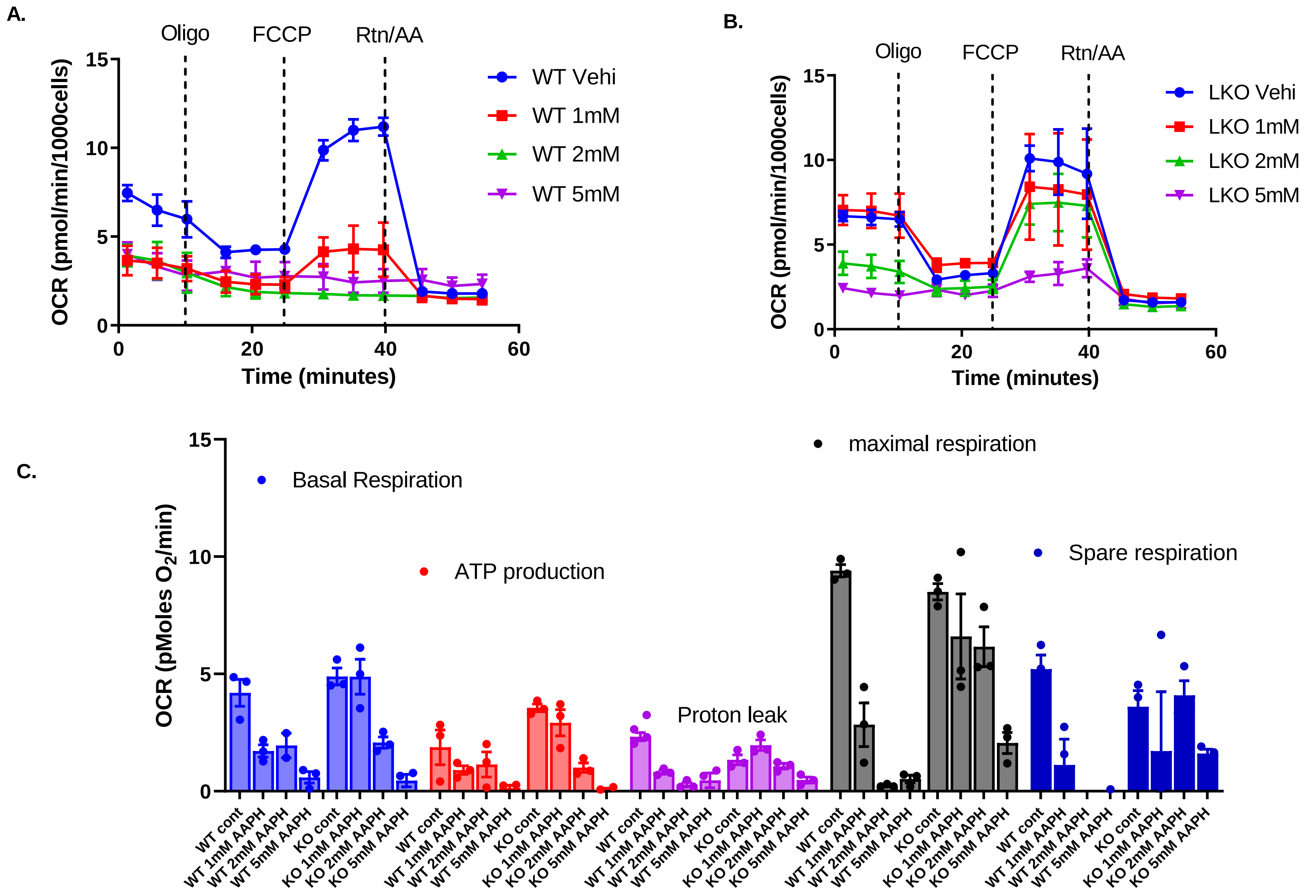

Figure S2. Mito-stress test_Dose response to AAPH: A and B, Traces of mito-stress test (glucose 10 mM, pyruvate 1 mM and L-glutamine 2 mM as substrates) using different respiratory chain inhibitors (oligomycin, FCCP and Rotenone + Antimycin) for different concentration from WT and LKO cells. **B,** Summary of basal respiration, maximal respiration, ATP production, proton leak and spare respiratory capacity from Seahorse XFe96 respectively (n=6 technical replicates/group).
